# Uniaxial Static Stress Estimation for Concrete Structures Using Digital Image Correlation

**DOI:** 10.3390/s19020319

**Published:** 2019-01-15

**Authors:** Junhwa Lee, Eun Jin Kim, Seongwoo Gwon, Soojin Cho, Sung-Han Sim

**Affiliations:** 1 School of Urban and Environmental Engineering, Ulsan National Institute of Science and Technology (UNIST), Ulsan 44919, Korea; lee.junhwa@unist.ac.kr (J.L.); ekim@unist.ac.kr (E.J.K.); ksw4430@unist.ac.kr (S.G.); 2 Department of Civil Engineering, University of Seoul, Seoul 02504, Korea; soojin@uos.ac.kr

**Keywords:** concrete, digital image correlation, stress relaxation method

## Abstract

This paper proposes a static stress estimation method for concrete structures, using the stress relaxation method (SRM) in conjunction with digital image correlation (DIC). The proposed method initially requires a small hole to be drilled in the concrete surface to induce stress relaxation around the hole and, consequently, a displacement field. DIC measures this displacement field by comparing digital images taken before and after the hole-drilling. The stress level in the concrete structure is then determined by solving an optimization problem, designed to minimize the difference between the displacement fields from DIC and the one from a numerical model. Compared to the pointwise measurements by strain gauges, the full-field displacement obtained by DIC provides a larger amount of data, leading to a more accurate estimation. Our theoretical results were experimentally validated using concrete specimens, demonstrating the efficacy of the proposed method.

## 1. Introduction

In full-scale civil engineering structures, the actual stress distribution due to self-weight and external loads allows a better understanding of the current structural status. For example, the monitoring of stress levels during construction can provide useful information for both design and construction verification, which can be helpful in assuring structural safety. In addition, the deterioration of prestressed concrete bridges, particularly due to loosened tendons, can be tracked by measuring the decreasing compressive stress in concrete girders [1,2,3]. Despite its usefulness and importance, the measurement of current stress levels in in-service concrete structures is not generally included in construction and maintenance practices [4]; it usually constitutes a challenging process, unless some force transducers were built in during the construction stage.

The stress relaxation method (SRM) [5] is one of the most practical and effective ways to measure the current stress in a structure. When applying the SRM, a small amount of damage is inflicted to the structure, inducing stress relaxation and its associated deformation around the damaged area. The stress level is estimated by analyzing the deformation redistributed by the damage. The SRM has been commonly adopted for the estimation of residual stresses in metals, in which it produces clear and consistent deformations following the inflicted damage. Several studies have applied the SRM to concrete structures, in which the deformation was typically measured using a few strain gauges [6,7,8]. However, concrete structures have considerable uncertainties, caused, for example, by inhomogeneity, inconsistent material properties, and difficulties in creating a hole in the concrete itself. Thus, deformation measurements made using strain gauges at a few points are insufficient to properly handle the uncertainties. Although several studies have shown the potential of measuring the static stress in concrete structures, the SRM has not been widely adopted in infrastructure maintenance, mainly due to the large uncertainty of concrete.

Previous studies involving the core-drilling method contained a couple of critical limitations that impeded the accurate measurement of stress in concrete structures. The core-drilling method involves the infliction of a relatively large amount of damage to the concrete structure, hence it is usually not the preferred method of practitioners and researchers. In literature, the size of the cores used for the SRM varied between 50 mm and 150 mm [7,8,9]; these holes can potentially behave as structural defects and are therefore not desired in practice. Another issue regarding previous studies is that insufficient information was collected about the deformation using the strain gauges. The strain gauge provides an average strain value, in correspondence with the gauge in a longitudinal direction, hence the available strain measurements were quite limited at several points. Particularly when the hole-drilling method with a small amount of damage is considered, the deformation field affects only a tiny area around the hole, which is difficult to measure using the strain gauges. The digital image correlation (DIC) technique was introduced to effectively address these issues.

Deformation measurements during the SRM can be enhanced by DIC [7,9,10] that provides the deformation field of an object’s surface. To perform the DIC, small and irregular speckles are densely painted on the object’s surface. Images of the speckle pattern are captured before and after deformation. A subset of the pattern is defined within the first image, then the location of the same subset is identified in the next image. By repeatedly finding the new location of subsets within the images, the two-dimensional displacement field can be obtained. DIC has been used to measure deformation for various purposes, including microscopic as well as large structures [11,12,13,14,15]. In addition, efforts have been made to apply DIC to concrete structures [16,17,18]. One of the advantages of DIC is that it can measure the displacement field from images; a capability which can very likely overcome the limitations of pointwise measurements performed using strain gauges. In some studies, DIC has been used to measure the deformation in concrete structures during the SRM. Trautner et al. [7] used DIC to measure the displacement fields caused by core drilling in concrete specimens. In their study, the use of the SRM combined with DIC resulted in a 10% error. Their results greatly contributed to the evaluation of in-situ stress in concrete structures, however, the diameter of the cores used (150 mm) impaired the applicability of their study to civil engineering structures. Indeed, high-performance stress estimation methods with practically allowable damages are desirable.

This study proposes a stress estimation method, based on a combination of stress relaxation and DIC. The proposed method initially measures the displacement field around a small drilled hole using DIC; this measurement is subsequently used to estimate the current stress level in the concrete structure by comparing the measured displacement field to that from a finite element model. DIC provides a full-field displacement at all points around the hole, which allows the collection of sufficient displacement information and an effective reduction in the measurement errors caused by the large uncertainty in concrete. The proposed method was validated through laboratory-scale experiments performed on concrete specimens.

## 2. Stress Estimation Method

**Figure 1:**
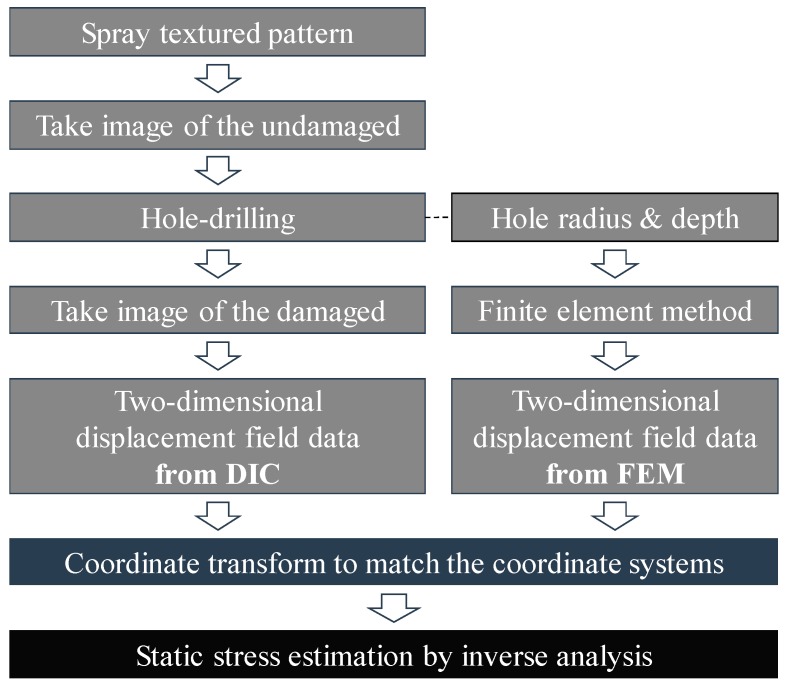
Flowchart of the algorithm used for the static stress estimation.

The proposed method introduces the DIC technique into the SRM, with the aim of estimating the static stress in large concrete structures. Rather than using a few strain or displacement data points, we used the two-dimensional displacement field obtained from the DIC, improving the stress estimation quality. As shown in Figure 1, the overall procedure of the proposed method consists of three steps: Obtaining two-dimensional displacement fields from the DIC and the finite element method (FEM), performing a coordinate transformation to have the same origin, and estimating the static stress. This section describes each step in detail.

Firstly, the two-dimensional displacement field data is obtained from the DIC: A Gaussian pattern, considered the best choice for the DIC technique [19], is applied to the concrete surface. Secondly, the static stress is relaxed by drilling a hole, leading to a redistribution of the displacement field. Lastly, the two-dimensional displacement field is acquired through DIC, by comparing the pattern before and after drilling the hole.

The reference displacement field, which becomes relaxed when the concrete is under a reference uniform stress, is generated through the FEM. Assuming that the concrete structure is within the linear elastic range, the amount of displacement relaxed by drilling the hole is linearly related to the amount of static stress. The product between the static stress and the reference displacement yields the same displacement field acquired by the DIC. Thus, the reference displacement field is established through the FEM, using the specimen information (e.g., the elastic modulus of the concrete, Poisson’s ratio, hole radius, and hole depth).

The next step is to match the origin of the coordinate system between the two displacement data sets. Because the only point shared by the DIC and the FEM is the hole center, the two coordinate systems are aligned to let the hole center be the common origin. In order to identify the hole center, the best-fitting circle is searched using a circular Hough transform [20]. The coordinates are then modified as follows:

(1) $$V_{FEM}(x^{\prime },y^{\prime })=V_{FEM}(x-x_{0},y-y_{0})$$

(2) $$V_{DIC}(x^{\prime },y^{\prime })=V_{DIC}(u-u_{0},v-v_{0})$$

where *V_FEM_* and *V_DIC_* are the displacement from the DIC and the FEM, (*x′*,*y′*) is the new coordinate system, (*x*,*y*) and (*u*,*v*) are the positions within the original coordinate systems, and (*x*_0_,*y*_0_) and (*u*_0_,*v*_0_) are the coordinates of the hole center in the coordinate system of the FEM and the DIC, respectively. Consequently, the static stress can be estimated by comparing those two displacement fields in the same coordinate system.

The final step is to estimate the stress, by comparing the DIC and the FEM in the same coordinate system. The two displacement results have a linear relationship:

(3) $$\frac{V_{DIC}(x^{\prime },y^{\prime })-V_{DIC}(0,0)}{\sigma }=\frac{V_{FEM}(x^{\prime },y^{\prime })-V_{FEM}(0,0)}{\sigma_{0}}$$

where *V_DIC_*(0,0) and *V_FEM_*(0,0) represent the displacement at the hole center, *σ* is the static stress to be estimated, and *σ*_0_ is a reference stress. The displacements from the DIC and the FEM are subtracted by *V_DIC_*(0,0) and *V_FEM_*(0,0), in order to disregard the rigid body motion of the whole specimen, as the displacement at the hole center represents the motion of the whole structural body. When the rigid body motion is neglected, *V_DIC_* equals *V_FEM_* but is amplified by the amount of static stress. Using the two-dimensional displacement fields (*V_DIC_* and *V_FEM_*), the static stress can be estimated as follows:

(4) $$\left\{\begin{array}{c} V_{DIC}(0,0)\\ \sigma /\sigma_{0} \end{array}\right\}={\left[\begin{array}{cc} 1 & V_{FEM}({x^{\prime }}_{1},{y^{\prime }}_{1})-V_{FEM}(0,0)\\ \vdots & \vdots \\ 1 & V_{FEM}({x^{\prime }}_{n},{y^{\prime }}_{n})-V_{FEM}(0,0) \end{array}\right]}^{+}\left\{\begin{array}{c} V_{DIC}({x^{\prime }}_{1},{y^{\prime }}_{1})\\ \vdots \\ V_{DIC}({x^{\prime }}_{n},{y^{\prime }}_{n}) \end{array}\right\}$$

where [·]^+^ is the Moore-Penrose inverse, (*x_k_’*,*y_k_’*) are the coordinates of the *k*-th point, and *n* is the number of points used. The optimal static stress of the concrete structure, denoted by *σ*, can hence be obtained.

## 3. Experimental Validation

**Figure 2:**
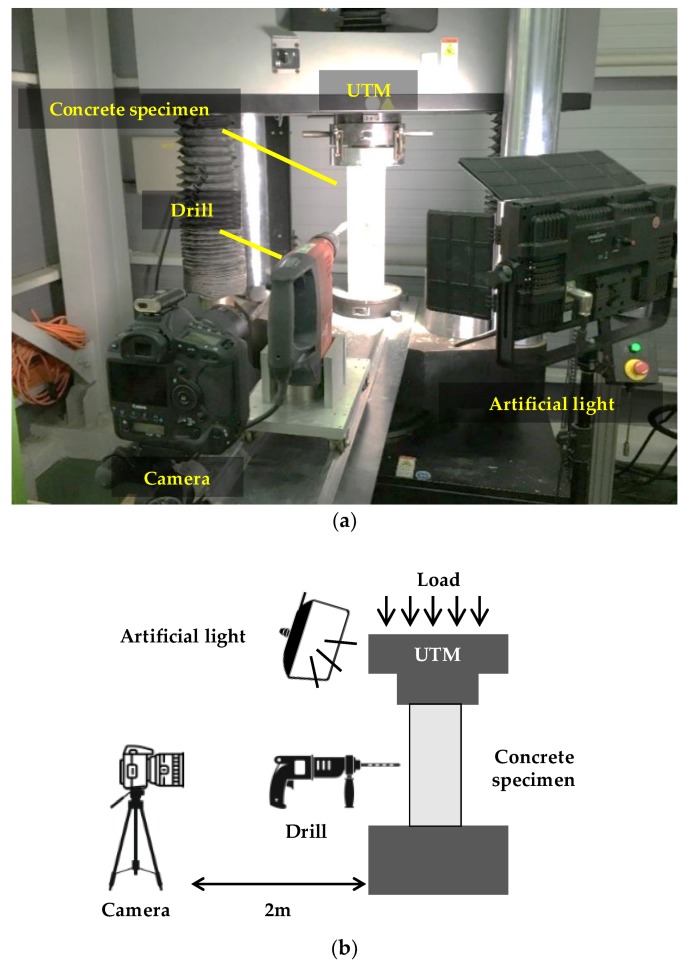
Laboratory-scale experiment: (**a**) Experimental setup; (**b**) Schematic view.

The proposed static stress estimation method is validated through laboratory-scale experiments. A total of five concrete specimens are prepared; their size, elastic modulus, and Poisson’s ratio are summarized in Table 1. The concrete specimens are compressed at approximately 14 MPa by the universal testing machine (UTM) shown in Figure 2. The imposed compressive stress is selected considering that the ultimate strength of the normal concrete specified in most design codes is 12–36 MPa [21,22]. The applied stress is measured using a load cell integrated within the UTM. An artificial light is placed near the concrete specimen to improve image contrast. A Canon EOS-1D camera equipped with a Canon EF 100 mm MACRO lens is placed at 2 m from the specimen, yielding a resolution of 0.08 mm/pixel. As DIC offers a subpixel resolution up to ±0.02 pixels, the displacement resolution of the camera configuration in this experiment is ±1.6 µm [23]. A hole with a 10 mm-radius and a 40-mm depth is drilled using a Hilti TE 50-AVR. For all five specimens, the stress estimated by the proposed method is compared with the applied stress. In this section, we first discuss the experimental results of the first test, and then describe the overall cases.

**Figure 3:**
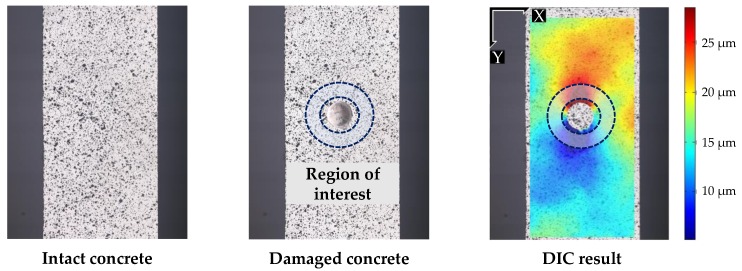
Displacement field from DIC using the two images before and after hole-drilling.

The displacement fields around the hole are calculated from the DIC. Ncorr [24] is adopted as a DIC tool to acquire two-dimensional displacement fields, using both the intact and the damaged images (Figure 3). The displacement field in the vertical direction is adopted in the calculation with the assumption that the target structure is in a state of uniform vertical stress. The smallest possible subset (radius = 79 pixels) is manually selected to avoid any discontinuity in the displacement field. The spacing between two consecutive subsets is set at 6 pixels, corresponding to 0.48 mm in the real specimen. Only the displacement field in the region of interest (see Figure 3) is considered in the calculation, due to the occurrence of erroneous DIC measurement in the proximity of the hole. The offset displacement, due to the rigid body motion, is calculated to be approximately 20 µm using Equation (4).

**Figure 4:**
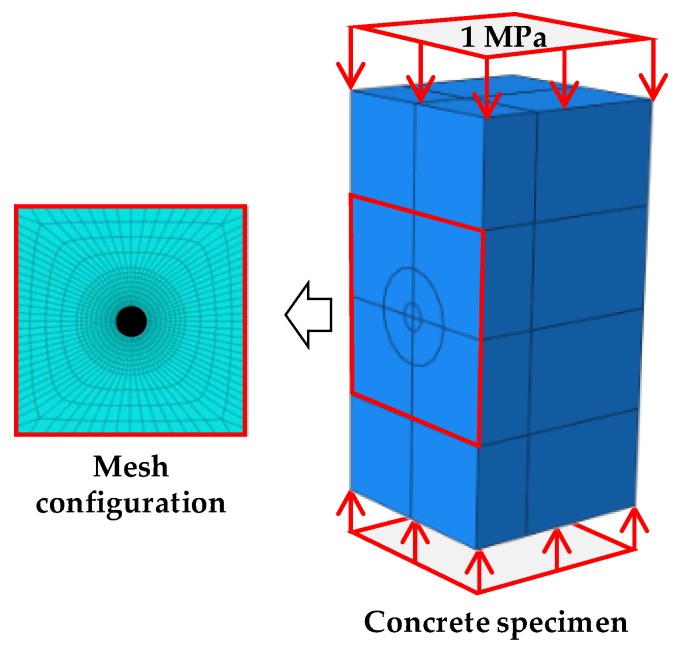
FE model of the concrete specimen.

**Figure 5:**
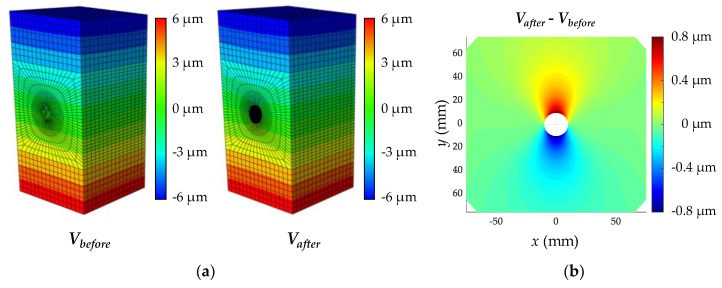
Displacement fields calculated using the FE model: (**a**) Displacement field of the intact and the damaged concrete, respectively denoted by *V_before_* and *V_after_*, obtained by the FE model; (**b**) The displacement field due to the hole-drilling.

The reference displacement is obtained using commercial FEM software, ABAQUS. A finite element (FE) model of the specimen shown in Figure 4 is built using the information described in Table 1. A mesh configuration, with a minimum size of 3.3 mm, is selected to accurately reproduce the displacement fields. The top and bottom faces are uniformly compressed by a reference stress (1 MPa) and a cylindrical shape (radius = 10 mm, depth = 40 mm) is removed in the meantime, to simulate the hole-drilling operation. Figure 5a shows the displacements of the FE models before and after drilling the hole. The difference between the maximum and minimum displacements relaxed by the drilling of the hole is about 1.6 µm; the typical shape of the displacement field due to the hole is shown in Figure 5b. The displacement field is then vectorized to form the matrix on the right side of Equation (4).

**Figure 6:**
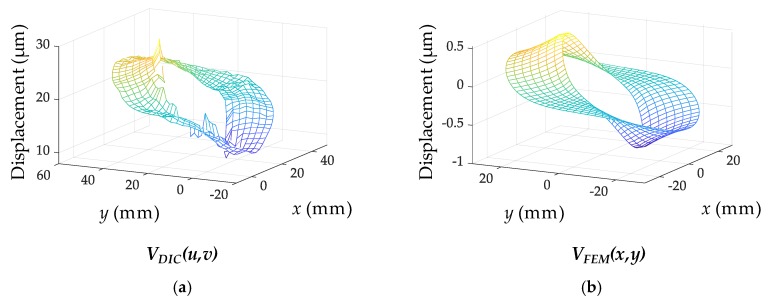
Displacement fields: (**a**) Obtained from DIC; (**b**) Obtained from ABAQUS.

The displacement fields obtained by the DIC and the FEM are shown in Figure 6. The shape of the resulting displacement field from the DIC resembles that from the FEM, whereas the magnitudes of the displacement fields and the origin locations are different between each other. The magnitudes and the origin locations are related to the amount of stress and the rigid body motion, respectively. Using Equation (4), the static stress and rigid body motion are estimated to be 15.53 MPa and 21.4 µm, respectively.

The experimental results are summarized in Table 2: They include the applied stress, the estimated stress, the estimation error, and the mean squared error (MSE) for the regression in Equation (4). Note that the MSE is not the estimation error but indicates the quality of the regression. The estimation error and MSE are defined as follows:

(5) $$error=\frac{\left|\sigma_{estimated}-\sigma_{applied}\right|}{\sigma_{applied}}\times 100$$

(6) $$MSE=\frac{1}{n}\sum_{k=1}^{n}{\left\{V_{DIC}^{\prime }(x_{k}^{\prime },y_{k}^{\prime })-V_{FEM}^{\prime }(x_{k}^{\prime },y_{k}^{\prime })\right\}}^{2}$$

(7) $$V_{DIC}^{\prime }(x^{\prime },y^{\prime })=V_{DIC}(x^{\prime },y^{\prime })-V_{DIC}(0,0)$$

(8) $$V_{FEM}^{\prime }(x^{\prime },y^{\prime })=\sigma_{estimated}\{V_{FEM}(x^{\prime },y^{\prime })-V_{FEM}(0,0)\}$$

where *σ_estimated_* and *σ_applied_* are the estimated and applied stress, respectively. The MSE represents the regression quality between the DIC and the FEM displacement fields, with smaller MSE values indicating more accurate stress estimations. Hence, the static stress can be estimated by using the proposed method and the estimation reliability can also be checked by evaluating the MSE.

The stress estimation results, shown in Table 2, can be visually verified by comparing the displacement fields from the FEM and DIC. To perform an effective comparison, *V_FEM_* and *V_DIC_* are adjusted using the estimated stress and rigid body motion in Equation (4), as follows:

(9) $$V_{FEM}^{\prime }=\frac{\sigma }{\sigma_{0}}V_{FEM}$$

(10) $$V_{DIC}^{\prime }=V_{DIC}-V_{DIC}(0,0)$$

**Figure 7:**
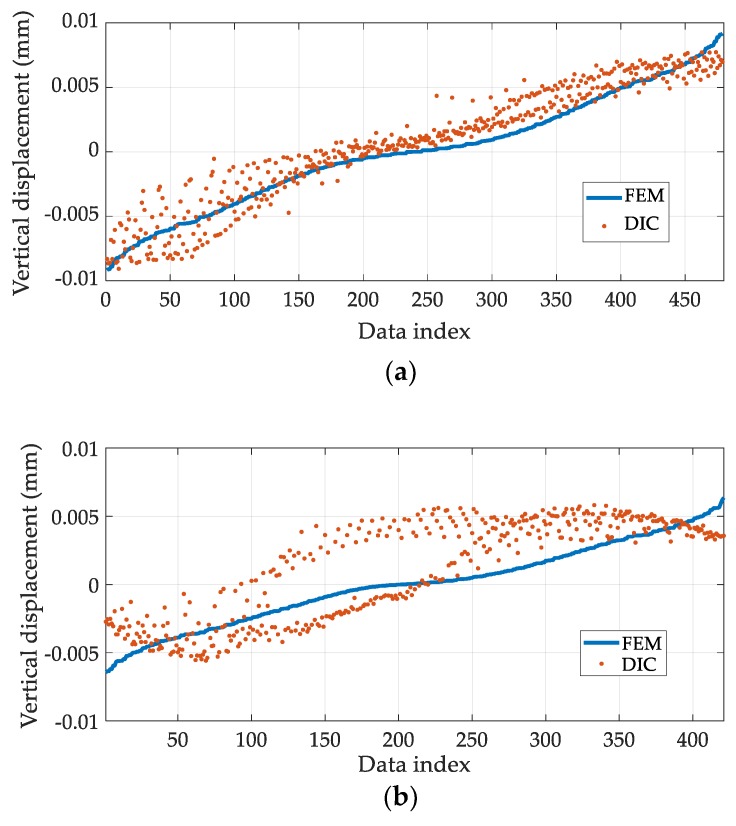
A pointwise comparison between the FEM results, scaled by the estimated stress and the DIC results, subtracted by the rigid body motion: (**a**) Test 1; (**b**) Test 5.

**Figure 8:**
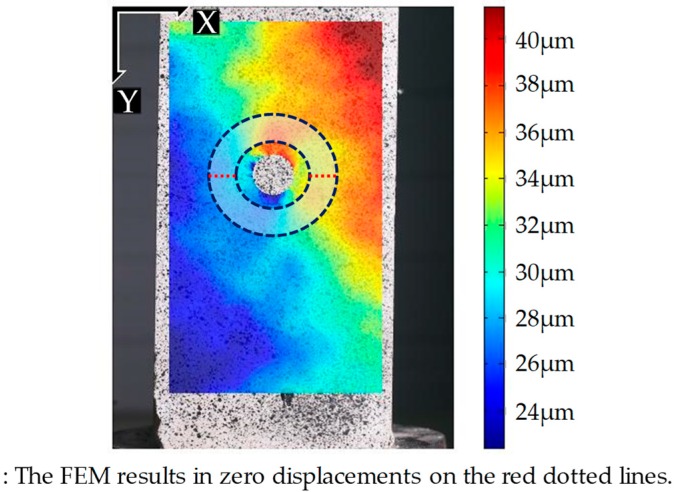
The displacement field obtained by the DIC for Test 5.

Figure 7 plots all the values in *V’_FEM_* and *V’_DIC_* with respect to their data index for Tests 1 and 5. During Test 1, the stress is more accurately estimated (the lowest MSE value) and the *V’_FEM_* and *V’_DIC_* datasets agree quite well with each other (Figure 7a). On the contrary, during Test 5, the *V’_DIC_* dataset deviates considerably from the *V’_FEM_* dataset, particularly between data indices 50 and 250; this deviation results in a large MSE value and in an inaccurate stress estimation (Figure 7b). The large deviation in the 50–250 index interval is caused by non-uniaxial stress conditions, due to the misalignment of the specimen. In fact, the top and bottom faces of the specimen are not perfectly parallel to each other, possibly due to the faulty fabrication, which causes biaxial stresses. In Figure 8, considerable displacement is observed on the red dotted line, whereas the corresponding displacement in the FE model is negligible. Thus, the actual stress distribution in the concrete specimen is expected to be quite different from *V_FEM_*, which is obtained by assuming uniaxial stress, resulting in a large MSE value for Test 5. Therefore, the quality of the stress estimation can be indirectly assessed using the MSE.

The proposed static stress estimation method has a few practical issues. Firstly, the hole-drilling points for stress estimation should be carefully selected to acquire important information about the structural integrity. Locations where structural members experience high stress levels would generally be a better option because the members can be vulnerable to the stress change and the measurement error has a smaller effect on the estimation. Furthermore, it is important to consider the possibility of unexpected cracks, caused by the hole-drilling process, which might distort the deformation pattern. Existent cracks should be identified, and corresponding crack regions should be removed from the displacement field before stress calculation. Following these precautions, the proposed method can be successfully applied to real concrete structures.

## 4. Conclusions

This study presented a uniaxial static stress estimation technique, which combines digital image correlation (DIC) and the stress relaxation method (SRM). The static stress in concrete structures can be released through the hole-drilling method. Firstly, the redistributed displacement field generated by the hole-drilling is measured through DIC. Secondly, a reference displacement field is calculated by using a finite element method (FEM), with given structural information. Lastly, the static stress is estimated through the least-square method, using the displacement fields from DIC and the FEM.

The proposed method was validated through laboratory-scale experiments on five concrete specimens, loaded by a universal testing machine (UTM). The best-fitting case resulted in a 5.67% larger stress, showing the smallest mean squared error (MSE) between the DIC and FEM displacement fields. The results with small MSE values provided better estimations than those with large MSE values. We conclude that the proposed method can be used to estimate the static stress in concrete structures; furthermore, the MSE values provide a quality check of the stress estimation.

## Figures and Tables

**Table 1:** Specimen information.

Concrete Specimen Size (Width × Height × Depth)	Type A: 100 mm × 400 mm × 100 mm
	Type B: 150 mm × 300 mm × 150 mm
Elastic modulus	24.4 GPa
Poisson’s ratio	0.17

**Table 2:** Stress estimation results.

Test Number	Specimen Type	Applied Stress (MPa)	Estimated Stress (MPA)	Estimation Error (%)	MSE (×10^−8^)
1	B	14.70	15.53	5.67	99.3
2	A	14.10	15.24	8.07	163.1
3	B	14.48	10.45	27.83	234.9
4	A	13.80	15.92	15.36	739.8
5	B	14.00	9.92	29.13	781.2

## Nomenclature

(*x*,*y*)	Position in the original coordinate system obtained by the FEM.
(*u*,*v*)	Position in the original coordinate system obtained by the DIC.
(*x*′,*y*′)	Position in the new coordinate system obtained by the FEM and DIC. The origin is the hole center.
(*x*_0_,*_y_*_0_)	Position of the hole center in the original coordinate system obtained by the FEM.
(*u*_0_,*v*_0_)	Position of the hole center in the original coordinate system obtained by the DIC.
*V_DIC_*(*x,y*)	Vertical displacement obtained by the DIC at (*x,y*).
*V_FEM_*(*x,y*)	Vertical displacement obtained by the FEM at (*x,y*).
*V’_FEM_*(*x,y*)	Adjusted vertical displacement obtained by the DIC at (*x,y*).
*V’_DIC_*(*x,y*)	Adjusted vertical displacement obtained by the FEM at (*x,y*).
